# Quality of Life of Mothers and Fathers of Children with Autism Spectrum Disorder in Jordan

**DOI:** 10.2174/17450179-v19-e230529-2022-40

**Published:** 2023-06-05

**Authors:** Eiman A. Ahmed, Sireen M. Alkhaldi, Hamza Alduraidi, Rania A. Albsoul, Mohammad Z. Alhamdan

**Affiliations:** 1 Department of Medicine Community, Ministry of Health, Sabha, Lybia; 2 Department of Family and Community Medicine, School of Medicine, The University of Jordan, Queen Rania Street, Amman 11942, Jordan; 3 Department of Community Health Nursing, School of Nursing, The University of Jordan, Queen Rania Street, Amman 11942, Jordan; 4 School of Medicine, The University of Jordan, Amman 11942, Jordan

**Keywords:** Quality of life, Parents, Autism spectrum disorder, WHOQOL – BREF, Children

## Abstract

**Background:**

Parenting children with autism spectrum disorder (ASD) is widely identified to be associated with life-long impairment in parents’ quality of life (QoL). However, there has been little information on the QoL of parents of children with ASD in the Jordanian context.

**Objective:**

This study aimed to assess the QoL among mothers and fathers who have children with ASD in Jordan and to identify factors associated with it.

**Methods:**

In this cross-sectional study, respondents were mothers and fathers of children with ASD attending autism rehabilitation centers in Amman. Data were collected from 206 participants using a validated questionnaire. Descriptive statistics, T-test, ANOVA and logistic regression, were applied.

**Results:**

Overall quality of life was low (mean= 2.32). The physical dimension scored the highest (mean =2.79), and the environmental dimension scored the lowest (mean= 2.06). Results indicated that fathers and parents with low education reported significantly lower QoL scores (p = .024 and 0.001, respectively).

**Conclusion:**

Among parents of children with ASD, parents at risk for low QoL were recognized. Our results can be utilized to design interventions to support mothers and fathers at risk in Jordan to enhance their QoL.

## 1 INTRODUCTION

Autism spectrum disorder (ASD) is a group of complex neurodevelopmental disabilities in children that manifests with increasing prevalence and recognition worldwide [1]. ASD is characterized by impairments in social interactions and communication, with restricted and repetitive behaviors, interests, and activities [2]. Moreover, recent research is exploring the relationship between autism and visual impairment, which impacts the quality of life of children [3]. It is called a spectrum because of the wide diversity in the presentation and severity of ASD, which makes it difficult to diagnose until the age of three years [4]. ASD diagnosis currently includes autistic disorder, pervasive developmental disorder-not otherwise specified (PDD-NOS), and Asperger syndrome [5]. There is no specific established cause of autism or real cure, although some behavioral interventions can improve children’s cognitive level and specific skills [6].

According to estimates from the CDC’s Autism and Developmental Disabilities Monitoring (ADDM) Network, the prevalence of ASD in the US increased from one in 150 in 2000 to one in 54 children under the age of eight (1.73% of the population) in 2016 [7, 8]. It has been estimated that the cost of supporting an individual with ASD during his/her lifespan in the US was $2.4 million, compared to $1.4 million for an individual without ASD [9]. The greatest cost components were special education services and parental productivity loss.

In the Middle East, experts estimate that the number of people with an autism spectrum disorder in Jordan ranged from 10,000 to 12,000 cases until 2017 [10]. The prevalence of ASD in Jordan is underestimated due to the social stigma associated with the disease, as it is still common for families to be embarrassed and hide their children with ASD from society [11-13]. Research on ASD in Jordan and the Arab world has accelerated in recent years, but it is still lagging behind Western countries [14, 15].

For parents, having a new child is considered stressful. However, when a child is born with a disability, parents find themselves burdened with long-lasting liability and demands that influence their lives and health [16-18]. Parent-mediated interventions are effective in improving children’s development [19]. Parents’ capacity to play a role in improving the health of their children with ASD will be particularly limited by their well-being and ability to cope. Research on the challenges and stress inherent in caring for a child with ASD has highlighted the impact on parents’ health and well-being, as not all parents cope effectively [16, 20, 21]. Globally, there is increasing awareness about ASDs and booming efforts to understand the life experiences of parents with children with ASD.

For parents of children with ASD, measuring the quality of life (QoL) provides a multidimensional useful way to comprehensively assess parents’ health and adaptation. QoL is an “individual’s perception of their position in life in the context of culture and value systems in which they live and concerning their goals, expectations, standards, and concerns” ’ [22]. The measurement of QoL has become essential for comprehending parents’ health outcomes and their predictors. QoL consists of an assessment of different domains of functioning, including physical, psychological, environmental, and social health [23]. Research on the QoL of parents of children with ASD and its associated factors remains sparse in terms of quantity and quality.

In the reviewed literature, studies have established a relationship between several factors and the QoL of parents who raise a child with autism spectrum disorder. Research has revealed that parental gender strongly impacts adaptation to a child’s disability. Some studies found that mothers have a higher burden of care, more physical health problems, and poorer QoL than fathers [21, 24-26], while other studies showed almost similar levels of QoL in mothers and fathers [27]. Parents with high education and higher income and socioeconomic status have reported higher QoL [12, 27-29]. In addition, parents who were employed (part-time or full-time) presented a significantly higher quality of life, and mothers who worked experienced better mental health compared to stay-at-home mothers [27, 30].

Regarding the severity of ASD, studies demonstrate that mothers, who perceived more child behavioral problems, had lower QoL [28, 31]. While the severity of ASD was associated with QoL in some studies [32, 33], other studies found no association between ASD severity and QoL [31]. In addition, parental stress has been associated with all domains of the QoL of parents caring for children with ASD in many studies [33, 34].

Previous studies have also shown that parents of older children have lower environmental QoL [33, 35], while Dardas *et al.* (2014) found better QoL among parents of older children [27]. Furthermore, the availability of social support contributes positively to parents’ QoL [21, 27, 28, 31], and other studies have reported an association between support from educational and rehabilitation services and parents’ QoL [30, 31, 36]. To the best of our knowledge, only two studies have examined the quality of life among parents of children with ASD in Jordan [27, 37]. In conclusion, there is a scarcity of research exploring the QoL of parents of children with ASD in Jordan, given the relatively high prevalence of ASD and its debilitating effects on these parents.

Therefore, this study aimed to assess various dimensions of QoL in mothers and fathers of children with ASD who attended private educational and rehabilitation centers in Jordan. It will answer the following research questions: What is the level of quality of life for mothers and fathers of children with ASD; whether there are any associations between QoL and the parents’ gender, age, marital status, and education; and which factors predict their QoL? This will be instrumental in identifying high-risk parents and highlighting the aspects of these parents that require reinforcement. This would inform clinicians and policymakers in Jordan of the real need for properly allocating resources for these families. The results of this study may help inform the design of effective interventions aimed at decreasing the caretaking burden on parents of children with ASD.

## 2 MATERIALS AND METHODS

This observational cross-sectional study aimed to assess the quality of life of mothers and fathers of children with autism spectrum disorder in Amman/Jordan. The target population of this study included parents of children with ASD attending private education and rehabilitation centers in Amman City. According to the Ministry of Social Development in Jordan, the total number of children in all autism centers was 392 in 10 centers in Amman city, according to the Ministry of Social Development in Jordan.

### 2.1 Study Population and Sampling

The sample size was calculated using the Steven Thompson equation to be equal to 195, with 10% of this sample (20 participants) added to compensate for non-response, resulting in a final sample size of 215. The selection of respondents from each center was proportional to the number of children in each center. Convenience sampling was used to select respondents from each center. The inclusion criteria were having autism disorder only and not accompanied by other disabilities.

### 2.2 Study Instrument

The World Health Organization Quality of Life Scale (WHOQOL – BREF) [22] is a validated questionnaire used to collect data. It consists of 31 items, covering two main sections. The first section had five paragraphs related to demographic characteristics (age, gender, education, marital status, and perceived health status). Current health status was measured as the self-perceived status of being ill or well at the time of the study. The second section has (26) items tailored to assess the four main domains of quality of life. The first is the physical domain, measured by questions 3, 4, 10, 15, 16, 17, and 18. An example of items in the physical domain is “To what extent do you feel that physical pain prevents you from doing what you need to do?”. The second is the psychological domain, measured by questions 5, 6, 7, 11, 19, and 26. An example of items in the psychological domain is “How much do you enjoy life?”. The third is the social domain, measured by questions 20, 21, and 22. An example of items in the social domain is “How satisfied are you with your personal relationships?”. Finally, the environmental domain was measured by questions 8, 9, 12, 13, 14, 23, 24, and 25. An example item in the environmental domain is “How safe do you feel with your daily life?”. Assessment of the study tool depended on a 5- point Likert scale ranging from 1-5 (strongly disagree to strongly agree) or from (very dissatisfied to strongly satisfied), where 5 stands for the highest quality of life. Back scoring was conducted for items 3 and 4 of the physical subscale and item 26 of the psychological subscale. Quality of life will be divided into three levels; we considered mean scores of 1-2.33 as low, 2.34-3.66 as moderate, and 3.67-5 as high. The category length that determines the level of importance (QoL levels) was calculated by subtracting the lower limit from the upper limit and dividing it by the number of levels.

Category length = (The upper limit of the alternative - the lower limit of the alternative) / Number of levels. = (5 -1) / 3 = 1.33.

The questionnaire was translated into Arabic and then back-translated into English to ensure the accuracy of vocabulary and language formulation. Pilot testing was performed on a random sample of 25 families with ASD children. They completed an Arabic version of the questionnaire to check whether the items were clear and understandable. Psychometric characteristics (validity and reliability) were examined. Pilot data were not included in the analysis.

The WHOQOL-BREF questionnaire is reliable; however, little consideration has been given to ensuring scale internal consistency and reliability regarding the context and population of interest. The Cronbach’s alpha coefficient for the total scale in this study was 0.88. Cronbach’s alphas for the physical, psychosocial, and environmental domains were 0.85, 0.84, 0.70, and 0.82, respectively. Test–Retest reliability was also applied, and a pilot sample of 25 families refilled the questionnaire 15 days after the first application. The results indicated a significant correlation in the responses of all 26 items in all domains between the first and second applications.

### 2.3 Data Collection Procedure

The researcher obtained a list of all education and rehabilitation centers for ASD in Amman, met the directors, and acquainted them with the study. Mothers and fathers who visited the facility at the time of data collection were invited to participate in the study, where only the father or mother of each child participated. Parents signed a written informed consent form and were assured of the privacy and confidentiality of all the data collected. Data were collected from the participating parents using a self-administered questionnaire. The data were collected from March to May 2019. Two hundred and fifteen questionnaires were distributed, and 206 participants completed the questionnaires, with a response rate of 96%. Five questionnaires were discarded due to incomplete answers and difficulty contacting parents after data collection, resulting in 201 questionnaires for analysis.

### 2.4 Ethical Considerations

IRB approval for this study was granted by the University of Jordan and the Jordan Ministry of Social Development (JMOSD). Confidence in the research data was ensured. After data collection, de-identified data were entered into SPSS and stored in secure files on the researcher’s computer with restricted access. The researcher stored hard copies of the completed data sheets in a locked file cabinet. The research team only used the data for research purposes.

### 2.5 Data Analysis

The researcher entered the data into (SPSS) version 21 and conducted data analysis. The significance level was set at (0.05) for all statistical analyses. Data analysis consisted of descriptive statistics (means, standard deviations, percentages) used to describe the study mothers’ and fathers’ characteristics and the quality of life domains. We used t-tests and ANOVA to verify the relationship between the four dimensions of QoL and demographic characteristics (age, sex, education level, marital status, and perceived illness). Binary logistic regression was performed to control for confounders and to assess the predictors and relative importance of various factors on quality of life after considering 2.5 as the cut-off point for high and low QoL.

## 3 RESULTS

Table **1** shows that 50.2% of the respondents were female, and 41.3% were 35–45 years old. Among all parents participating, 65.2% held bachelor’s degrees, and 18.9% had high school or lower levels of education. More than 80% of the respondents were married. Approximately one-quarter of the parents (27.4%) perceived themselves as ill during data collection. Most parents (74.2%) evaluated their quality of life as poor or very poor, and more than 65% were dissatisfied or very dissatisfied with their health.

### 3.1 QoL Overall and Domain Scores

The overall QoL score (out of 5) was calculated for each participant, with a mean of 2.32 out of 5, which is considered low (the borderline between low and moderate). In addition, a separate score out of five was calculated for each scale domain: physical, psychological, environmental, and social QoL. The mean scores are shown in Table **2**. The physical QoL domain had the highest mean score (mean= 2.79), indicating a moderate level of quality of life. The Environmental QoL domain had the lowest score (mean= 2.06), suggesting a low level.

### 3.2 Association between Socio-demographic Characteristics and QoL Scores

Our results show that fathers of children with autism had a significantly lower mean overall QoL score than mothers (t = -3.227, p = .001). Likewise, fathers scored significantly lower than mothers in each of the four domains of the QoL scale: physical (t = -3.846, p = .000), psychological (t = -2.072, p = .040), environmental (t = -2.634, p = .009), and social (t = -2.282, p = .024). In addition, a statistically significant association was found between educational level and QoL overall score (F = 20.771, p = .000), as well as each of the four domains of the QoL scale: physical (F = 3.852, p = .010), psychological (F = 13.606, p = .000), environmental (F = 15.646, p = .000), and social (F = 17.568, p = .000). Parents with higher education had significantly higher scores. However, age group, marital status, and perceived illness were not significantly associated with QoL overall or domain scores (Table **3**).

A cutoff score of 2.5 out of 5 was used to divide the sample members into low (n = 143, 71.1%) and high (n = 58, 28.9%) QoL groups to use binary logistic regression. After checking all assumptions, a binary logistic regression model was used to test the effect of a combination of independent variables (gender, age group, marital status, educational level, and perceived illness) on the cutoff overall QoL score (*i.e.*, low *vs.* high QoL). Table **4** presents the logistic regression results. The model was statistically significant, Chi-square (df) = 29.466 (5), p = .000. The five combined independent variables were able to explain 19.5% of the variance in the cutoff overall QoL score (Nagelkerke R^2^ = .195). Two of the five independent variables had statistically significant unique contributions to the model: gender (p = .042) and educational level (p = .001). Mothers had 2.031 more odds of having a high overall QoL than fathers, and parents with graduate education had 2.778 more odds of having a high overall QoL compared to parents with lower educational levels. The remaining three independent variables (age, marital status, and perceived illness) were not statistically significant (Table **4**).

## 4 DISCUSSION

Having children with autism poses a considerable lifelong burden to their families. This burden negatively influences the health and QoL of parents, which diminishes their ability to care for other children and their families. Understanding the quality of life of mothers and fathers who have children with autism and factors that contribute to their QoL may help guide interventions designed to alleviate this burden. Therefore, this exploratory, descriptive cross-sectional study aimed to assess various dimensions of QoL of mothers and fathers of children with ASD in Jordan, investigate some of the factors associated with their QoL, and explore predictors of their QoL.

### 4.1 Quality of Life of Parents of Children with ASD

Our findings indicate a low quality of life for mothers and fathers of children with autism spectrum disorder in Jordan. The results suggested that both mothers and fathers demonstrated the lowest QoL in the environmental domain (mean= 2.06), followed by the social domain (mean= 2.18) while having a better quality of life in the physical domain (mean= 2.79). This can be explained by the fact that daily family activities are centered on the needs and demands of children. Outside the routine home environment with a child with ASD and special needs is highly challenging because of the limited availability of an appropriate, safe, healthy physical environment.

Moreover, there is a scarcity of social relationships that parents can maintain due to the never-ending relentless care required by the child. This is consistent with the results of studies from Jordan [37], Poland [26], and Greece [38], where the environmental aspects of QoL were lowest among parents of children with ASD. Perumal *et al.* in India investigated the QoL of families of children with ASD and found the social and environmental domains to be severely impaired [39]. Research by Pineo *et al.* showed that parents of children with ASD had better physical health and the lowest social relationship parameters [38]. In contrast, the physical domain of QoL was the most adversely affected in other studies conducted in Japan [24], Bahrain [40], and Taiwan [33]. The findings of Ansari *et al.* indicated that mothers of children with ASD reported high physical health aspects of QoL [40]. Moreover, Shattnawi *et al.* explored mothers’ everyday experiences of raising a child with ASD. They showed that family relationships and social life were jeopardized and posed one of their major life challenges [18].

### 4.2 Determinants and Predictors of the QoL of Parents of Children who have ASD

The findings of this study demonstrate that mothers experience significantly better QoL than fathers do. This is inconsistent with a study in Jordan [37] that found no difference between mothers and fathers. In addition, in most reviewed studies, mothers had lower scores than fathers in most aspects of quality of life, which is logical and expected, since mothers are the primary caregivers of children [21, 24-26, 31, 38]. The higher QoL among mothers in this sample can be explained by the fact that children receive rehabilitation and education in stay-in specialized centers, which alleviates a large portion of the burden of caregiving on the mother. This cannot be generalized to the mothers of children with ASD in Jordan, especially those who cannot afford expensive rehabilitation center services and instead attend governmental facilities or do not receive any services at all.

A higher socioeconomic status, mainly education, indicated an increased ability to utilize resources and specialized care available in the community. It is also frequently reported to positively impact people’s quality of life. The current study revealed that level of education, as the other most important measure of socioeconomic status, was significantly associated with parents’ QoL. This finding aligns with some studies in the literature [38, 41]. Nevertheless, Dardas and Ahmad found no association between parents’ education and QoL in Jordan [37]. Despite their high levels of education, they found low levels of employment among mothers of children with ASD. This may be due to the fact that the children’s disruptive behavior requires mothers to stay at home to provide the necessary care.

In this study, no data were collected on income level, which is an important measure of socioeconomic status [41]. This was because the respondents in this study were high-income parents of children with ASD who attended private rehabilitation centers that cost the US $1000- 1500 per month. This is in line with the findings of Al-Khateeb *et al.* (2019) in their review, which revealed that the majority of participants in studies of autism were from middle- and high-income families. This may be explained by the fact that a sample of parents raising children with ASD is more conveniently accessible through the rehabilitation and educational centers that their children attend.

This study has contributed considerably to the limited body of research in Jordan and the region on the quality of life of parents of children with ASD, highlighting their immediate and considerable need for support and services. Nonetheless, this study had certain limitations to be addressed when examining the results. The cross-sectional design, with a single measurement of exposures and outcomes, makes it difficult to derive causal associations. Information about children’s age, degree of disability, and family income level is lacking; therefore, their effect on mothers’ QoL could not be taken into account in this study. As participation was restricted to parents of children who received care in private rehabilitation centers, the results of this study cannot be generalized to parents of children with ASD in Jordan.

Parents of children with ASD need to prioritize psychosocial interventions specifically attuned to their needs [42], which may include family-to-family support. Jamison *et al.* (2017) found that the family peer advocate (FPA) model significantly increased knowledge about ASD and reduced stress for caregivers [43]. Peer support interventions can positively reflect their parenting confidence, mental health, and ability to care for and provide for the whole family [40, 44, 45]. Community-level interventions are necessary to raise awareness and improve the perception of autism. This will encourage social support for these parents, which will play a crucial role in enhancing their QoL [28, 46]. This may help minimize the stigma related to the disorder, which isolates children and their parents from society. Additionally, interventions may include financial aid for low-income families to compensate for the income lost from raising a child with ASD. Future research may evaluate support services available for parents of children with ASD, both governmental and non-governmental, investigating gaps with present opportunities for enhancement. Further research is necessary to understand the correlations between the QOL of parents of children with ASD in Jordan.

## CONCLUSION

In conclusion, the findings of this study have called for the debilitating effects of raising a child with autism on the QoL of mothers and fathers. It has also highlighted the protective role of education, especially for mothers, in maintaining the health and QoL of families and, consequently, of children with ASD.

## Figures and Tables

**Table 1:** Socio-demographic characteristics of respondents in the study of quality of life of families with (ASD) children in Amman in 2018 (N=201).

** Variables **	**Category**	**Frequency**	**Percent (%)**
** Gender**	Fathers	100	49.8
	Mothers	101	50.2
**Age**	<35 years	67	33.3
	35 – 45 years	83	41.3
	>45 years	51	25.4
**Education**	High school or less	38	18.9
	Diploma	14	7.0
	Bachelor	131	65.2
	Higher Education	18	9.0
**Marital Status**	Married	161	80.1
	Separated	28	13.9
	Widower	12	6.0
**Perceived Illness**	Ill	55	27.4
	Healthy	146	72.6
**Self-rated quality of life**	Very poor	97	48.3
	Poor	52	25.9
	Neither poor nor good	27	13.4
	Good	21	10.4
	Very good	4	2.0
**Perceived satisfaction with own health**	Very dissatisfied	51	25.4
	Dissatisfied	80	39.8
	Neither satisfied nor dissatisfied	27	13.4
	Satisfied	26	12.9
	Very satisfied	17	8.5

**Table 2:** Mean QoL overall and domain scores (N = 201).

**QoL Domain**	**Range**	**Mean**	**SD**	**Level ^b^**
**Overall QoL^a^**	(1.49 – 4.26)	2.32	.60	low
**Physical QoL^a^**	(1.83 – 4.83)	2.79	.55	moderate
**Psychological QoL^a^**	(1.17 -4.33)	2.26	.63	low
**Environmental QoL^a^**	(1.00 – 4.50)	2.06	.71	low
**Social QoL^a^**	(1.00 – 5.00)	2.18	1.08	low

**Note:** ^a^Score out of 5.

^b^Levels are:1-2.33= low, 2.34-3.66= moderate and 3.67-5= high.

**Table 3:** Association between socio-demographic characteristics and QoL overall and domain Scores (N = 201).

**Socio-demographic Variable**		**Overall QoL^a^** mean (SD)	**Physical QoL^a^** mean (SD)	**Psychological QoL^a^** mean (SD)	**Environmental QoL^a^** mean (SD)	**Social QoL^a^** mean (SD)
**Gender**	**Fathers **(n = 101)	2.19 (.53)	2.65 (.44)	2.17 (.59)	1.93 (64)	2.01 (.98)
	**Mothers **(n = 100)	2.46 (.65)	2.94 (.61)	2.35 (.65)	2.19 (.76)	2.35 (1.14)
	**t (df)**	-3.227 (199)	-3.846 (199)	-2.072 (199)	-2.634 (199)	-2.282 (199)
	** p **	.001*	.000*	.040*	.009*	.024*
**Age**	**< 35 years **(n = 67)	2.33 (.61)	2.80 (.54)	2.27 (.57)	1.99 (.67)	2.26 (1.11)
	**35-45 years **(n = 83)	2.34 (.61)	2.81 (.56)	2.24 (.68)	2.11 (.78)	2.20 (1.05)
	**> 45 years **(n = 51)	2.28 (.60)	2.76 (.56)	2.29 (.62)	2.05 (.67)	2.02 (1.07)
	**F (df)**	.157 (2, 198)	.128 (2, 198)	.136 (2, 198)	.529 (2, 198)	.734 (2, 198)
	** p **	**.854**	**.880**	**.873**	**.590**	**.481**
**Social Status**	**Married **(n = 161)	2.29 (.45)	2.78 (.57)	2.24 (.58)	2.03 (.69)	2.11 (1.03)
	**Separated **(n = 28)	2.46 (.71)	2.77 (.41)	2.37 (.82)	2.13 (.78)	2.56 (1.32)
	**Widow/er **(n = 12)	2.43 (.70)	2.97 (.60)	2.33 (.70)	2.19 (.93)	2.22 (1.00)
	**F (df)**	1.097 (2, 198)	.694 (2, 198)	.601 (2, 198)	.419 (2, 198)	2.130 (2, 198)
	** p **	**.336**	**.501**	**.549**	**.658**	**.122**
**Education**	**≤ High school **(n = 38)	2.04 (.51)	2.59 (.44)	2.07 (.54)	1.79 (.70)	1.73 (.80)
	**Diploma **(n = 14)	2.17 (.75)	2.76 (.63)	2.12 (.46)	1.91 (.86)	1.88 (1.37)
	**Bachelor **(n = 131)	2.29 (.50)	2.81 (.54)	2.22 (.55)	2.02 (.59)	2.13 (.96)
	**Graduate **(n = 18)	3.21 (.60)	3.11 (.64)	3.06 (.85)	3.01 (.76)	3.67 (.96)
	**F (df)**	20.771 (3, 197)	3.852 (3, 197)	13.606 (3, 197)	15.646 (3, 197)	17.568 (3, 197)
	** p **	.000*	.010*	.000*	.000*	.000*
**Perceived Illness**	**Ill **(n = 55)	2.26 (.61)	2.74 (.56)	2.21 (.64)	2.05 (.74)	2.04 (1.07)
	**Healthy **(n = 146)	2.35 (.60)	2.81 (.55)	2.28 (.62)	2.06 (.71)	2.23 (1.08)
	**t (df)**	-.936 (199)	-.814 (199)	-.776 (199)	-.143 (199)	-1.140 (199)
	** p **	**.350**	**.416**	**.439**	**.887**	**.256**

^a^Score out of 5.

* Statistically significant: p < .05.

**Table 4:** Binary Logistic Regression of Gender, Age Group, Marital Status, Educational Level, and Illness on Cutoff Overall QoL Score (N = 201).

**Independent Variable ^b^**	**B**	**Wald**	***df***	***p***	**Odds Ratio**	**95% CI**	
						**Lower limit**	**Upper limit**
**Gender **(Fathers) Mothers	0.709	4.129	1	0.042^c^	2.031	1.025	4.025
**Age Group **(<35 years) ≥35 years	0.180	0.269	1	0.604	1.197	0.607	2.364
**Marital Status **(Not married) Married	0.520	3.030	1	0.082	1.681	0.937	3.018
**Educational Level **(<Graduate education) Graduate Education	1.022	15.89	1	0.000^c^	2.778	1.681	4.591
**Perceived Illness **(Healthy) Ill	0.132	0.108	1	0.743	1.141	0.519	2.508

**Note:** ^a^Model’s chi-square *(df)* = 29.466 *(5),* *p* = .000, Nagelkerke R^2^ = .195.

^b^Reference category in parenthesis.

^c^Statistically significant, p < .05

## Data Availability

The data supporting the findings of the article is available in the Zenodo at https://zenodo.org/record/7852343#.ZEJz L3ZBw2w.

## LIST OF ABBREVIATIONS

JMOSD: = Ordan Ministry of Social Development
ASD: = Autism Spectrum Disorder
ADDM: = Autism and Developmental Disabilities Monitoring
